# 3D Chitin Scaffolds of Marine Demosponge Origin for Biomimetic Mollusk Hemolymph-Associated Biomineralization *Ex-Vivo*

**DOI:** 10.3390/md18020123

**Published:** 2020-02-19

**Authors:** Marcin Wysokowski, Tomasz Machałowski, Iaroslav Petrenko, Christian Schimpf, David Rafaja, Roberta Galli, Jerzy Ziętek, Snežana Pantović, Alona Voronkina, Valentine Kovalchuk, Viatcheslav N. Ivanenko, Bert W. Hoeksema, Cristina Diaz, Yuliya Khrunyk, Allison L. Stelling, Marco Giovine, Teofil Jesionowski, Hermann Ehrlich

**Affiliations:** 1 Faculty of Chemical Technology, Institute of Chemical Technology and Engineering, Poznan University of Technology, Berdychowo 4, 60965 Poznan, Poland; tomasz.g.machalowski@doctorate.put.poznan.pl (T.M.); teofil.jesionowski@put.poznan.pl (T.J.); 2 Institute of Electronics and Sensor Materials, TU Bergakademie Freiberg, Gustav-Zeuner str. 3, 09599 Freiberg, Germany; iaroslavpetrenko@gmail.com; 3 Institute of Materials Science, TU Bergakademie Freiberg, 09599 Freiberg, Germany; schimpf@iww.tu-freiberg.de (C.S.); rafaja@ww.tu-freiberg.de (D.R.); 4 Clinical Sensoring and Monitoring, Department of Anesthesiology and Intensive Care Medicine, Faculty of Medicine, TU Dresden, 01307 Dresden, Germany; roberta.galli@tu-dresden.de; 5 Faculty of Veterinary Medicine, Department of Epizootiology and Clinic of Infectious Diseases, University of Life Sciences, Głęboka 30, 20612 Lublin, Poland; achantina@op.pl; 6 Faculty of Medicine, University of Montenegro, Kruševac bb, 81000 Podgorica, Montenegro; snezap@ac.me; 7 Department of Pharmacy, National Pirogov Memorial Medical University, 21018 Vinnitsa, Ukraine; algol2808@gmail.com; 8 Department of Microbiology, National Pirogov Memorial Medical University, 21018 Vinnitsa, Ukraine; valentinkovalchuk2015@gmail.com; 9 Department of Invertebrate Zoology, Biological Faculty, Lomonosov Moscow State University, 119992 Moscow, Russia; ivanenko.slava@gmail.com; 10 Taxonomy and Systematics Group, Naturalis Biodiversity Center, 2333CR Leiden, The Netherlands; bert.hoeksema@naturalis.nl; 11 Groningen Institute for Evolutionary Life Sciences, University of Groningen, 9747AG Groningen, The Netherlands; 12 Harbor Branch Oceanographic Institute, Florida Atlantic University, 5600 Old Dixie Hwy, Fort Pierce, FL 34946, USA; taxochica@gmail.com; 13 Department of Heat Treatment and Physics of Metal, Ural Federal University, Mira Str. 19, 620002 Ekaterinburg, Russia; juliakhrunyk@yahoo.co.uk; 14 The Institute of High Temperature Electrochemistry of the Ural Branch of the Russian Academy of Sciences, Akademicheskaya Str. 20, 620990 Ekaterinburg, Russia; 15 Department of Biochemistry, Duke University Medical School, Durham, NC 27708, USA; antistokes@gmail.com; 16 Department of Sciences of Earth, Environment and Life, University of Genoa, Corso Europa 26, 16132 Genova, Italy; mgiovine@unige.it; 17 Center for Advanced Technology, Adam Mickiewicz University, 61614 Poznan, Poland

**Keywords:** chitin, scaffold, sponges, hemocytes, hemolymph, biomineralization, calcite

## Abstract

Structure-based tissue engineering requires large-scale 3D cell/tissue manufacture technologies, to produce biologically active scaffolds. Special attention is currently paid to naturally pre-designed scaffolds found in skeletons of marine sponges, which represent a renewable resource of biomaterials. Here, an innovative approach to the production of mineralized scaffolds of natural origin is proposed. For the first time, a method to obtain calcium carbonate deposition ex vivo, using living mollusks hemolymph and a marine-sponge-derived template, is specifically described. For this purpose, the marine sponge *Aplysin aarcheri* and the terrestrial snail *Cornu aspersum* were selected as appropriate 3D chitinous scaffold and as hemolymph donor, respectively. The formation of calcium-based phase on the surface of chitinous matrix after its immersion into hemolymph was confirmed by Alizarin Red staining. A direct role of mollusks hemocytes is proposed in the creation of fine-tuned microenvironment necessary for calcification ex vivo. The X-ray diffraction pattern of the sample showed a high CaCO_3_ amorphous content. Raman spectroscopy evidenced also a crystalline component, with spectra corresponding to biogenic calcite. This study resulted in the development of a new biomimetic product based on ex vivo synthetized ACC and calcite tightly bound to the surface of 3D sponge chitin structure.

## 1. Introduction

Modern structure-based tissue engineering [1] urgently requires large-scale 3D cell/tissue-manufacture technologies, to create biologically active 3D scaffolds. Consequently, 3D scaffolds must possess biological, immunological, physicochemical, structural and mechanical cues at nanoscale, microscale and macroscale levels. Mostly artificially fabricated or prefabricated scaffolds must be bio- and eco-compatible [2] and should be suitable to support optimal cell growth, differentiation and proliferation [3], with the final goal of developing corresponding soft and hard tissues on request.

Recent focus in biomedicine has been concentrated on non-artificial, naturally prefabricated [4] skeletal structures of marine origin [5,6,7,8,9,10,11] that serve as examples of “marine biomimetics” [12]. This trend is well represented in the book by Choi and Ben-Nissan entitled *Marine-Derived Biomaterials for Tissue Engineering Applications* [13]. We completely agree with the statement of Professor Pierfrancesco Morganti that “the use of natural polymers in substitution to the petrol-derived ones seems the best way to produce skin-friendly healthy tissues and, slowing down the increased plastics production and waste, to try to save the Earth’s environment equilibrium and biodiversity” [2].

Special attention is currently paid to naturally pre-designed scaffolds found in skeletons of diverse marine sponges, which represent unique renewable resources for the sustainable development of novel 3D biomaterials [14,15,16,17,18,19]. The main players in this field are marine demosponges (phylum Porifera: class Demospongiae) of the orders Dictyoceratida (subclass Keratosa) and Verongiida (subclass Verongimorpha), which produce microporous proteinaceous (spongin)-based [16,20,21,22,23] and chitin-based [23,24,25,26,27,28,29,30,31,32,33,34] 3D skeletal constructs, respectively. Traditionally, it is mostly arthropods’ chitin that is recognized as the biological material suitable for application in biomedicine (for review, see [35,36,37,38,39]). Due to its biocompatibility, chitin is applied in dermocosmetics [2,40,41,42,43] and as wound-dressing biomaterial [44,45,46]. Therefore, reports concerning the successful clinical application of chitin in the form of dressings and membranes [47,48,49,50,51] are of crucial interest. Recently, Fang et al. [52] reported a method for producing 3D wound-dressing sponges made of quaternary chitin/partially deacetylated chitin nanofibers. Obtained material exhibited instant water/blood-triggered expansion and superabsorbent capacity. Moreover, authors proved such chitinous 3D sponges attract and stimulate blood cells/platelets, promoting blood coagulation and display superior hemostatic performance to traditional hemostatic materials [52].

**Figure 1:**
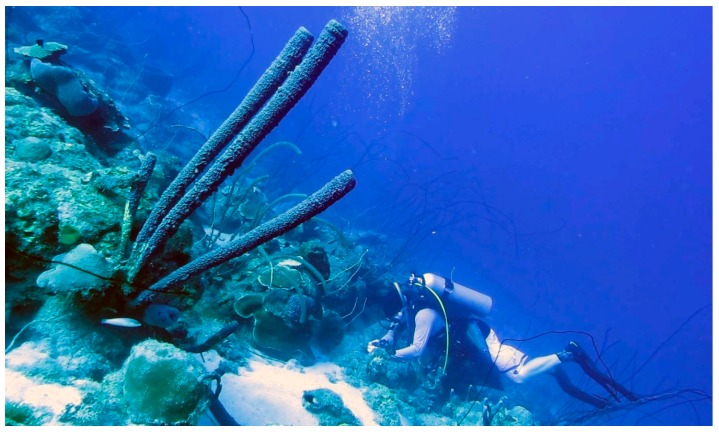
The giant Caribbean stove-pipe sponge *Aplysina archeri* (Demospongiae, Verongiida: Aplysinidae) produces up to 1.5-m-long skeletal tubes (of inner diameter ≤10 cm) made of mineralized chitin [4]. This image was made by V.N. Ivanenko, on June 12, 2017, in the coastal waters of Curaçao (Playa Marie Pampoen; 12°05’24″N, 68°54’19″W; depth 21 m).

The discovery of chitin in the skeleton of Cambrian demosponge *Vauxia gracilenta* (Walcott, 1920) [53], closely related to verongiids, confirmed a million-years-long success of sponges belonging to the order Verongiida, with respect to naturally optimized architecture of their chitinous, robust, fiber-based, microporous skeletons. The content of chitin in verongiids is species-dependent and ranges between 5% and 70% [4,24,27,29,30]. One of the most important advantages of sponge chitin, in contrast to traditionally used chitin sources, such as fungi [54], coralline algae [55], mollusks [56], corals [57], insects, spiders and crustaceans [58,59,60,61], is the size of sponges: they can reach up to 2 m in diameter (i.e., the elephant ear sponge *Ianthella basta* (Pallas, 1766)) [27,62], or up to 1.5 m length, as with the Caribbean stove-pipe sponge *Aplysina archeri* (Higgin, 1875) used in this study (Figure 1). Furthermore, verongiid sponges can be successfully cultivated under marine farming conditions [63,64,65,66,67] and possess the unique ability to regenerate their skeletal tissues up to 12 cm per year [62].

Following the discovery of chitin content in sponges in 2007 [68,69], the first reports on the applications of 3D sponge-derived chitin scaffolds in tissue engineering appeared in 2010 [25,70]. Our scaffolding strategy [53,71] is based on the application of naturally prefabricated 3D chitinous scaffolds of poriferan origin as one of the forms of decellularized matrices [72]. Previously, we reported that the decellularized chitinous 3D matrices of the verongiid sponges *Aplysina aerophoba* [73,74,75] and *Ianthella basta* [76,77] can be applied in tissue engineering of selected human bone-marrow-derived mesenchymal stromal cells (hBMSCs) and human dermal cells MSCs (for overview, see [77]). Human chondrocytes [25,70], as well as cardiomyocytes [34], also showed good growth and proliferation features in experiments using 3D chitinous scaffolds isolated from the cultivated demosponge *A. aerophoba*.

**Figure 2:**
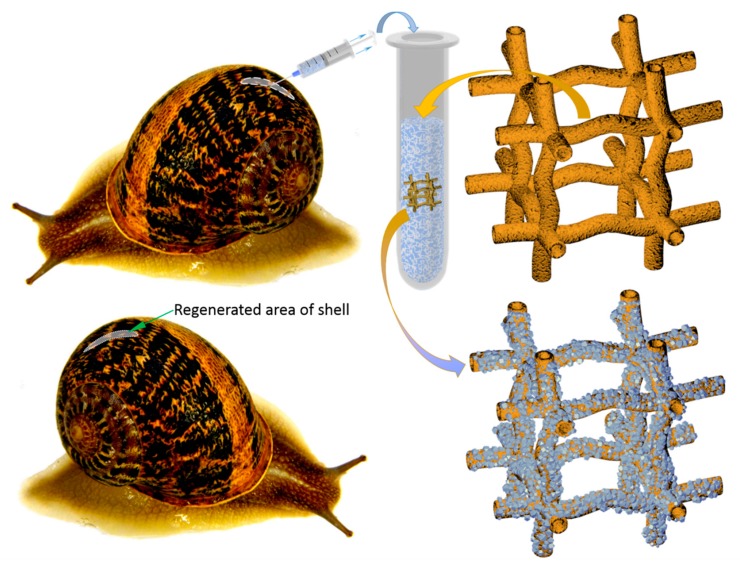
Principal schematic view of non-lethal manual extraction of *C. aspersum* hemolymph and its application for ex vivo biomineralization, using 3D chitinous scaffolds isolated from sponges with respect to obtain both amorphous and microcrystalline calcium carbonate phases (see also Figure 14).

It is well recognized that special multifunctional cells circulating in molluscan hemolymph, known as hemocytes, are crucial players in mollusk shell formation and repair processes [78,79,80,81]. These cells are responsible not only for calcium and bicarbonate ions transport but, due to a suitable microenvironment, also for biomineral crystal formation directly in the cell [82]. Consequently, such observations allowed us to create a methodological trend based on the development of calcium carbonate crystals deposition ex vivo by living mollusks hemolymph, using external templates [80,83,84,85]. For this purpose, both 3D chitinous scaffolds from *A. archeri* sponge and terrestrial snail *Cornu aspersum* (O.F. Müller, 1774) (previously known as *Helix aspersa*, (Mollusca: Gastropoda: Stylommatophora: Helicidae) (Figure 2) were used as an appropriate matrix and as the hemolymph donor, respectively. This mollusk is used as escargot meat and slime for cosmetic applications; thus, mollusks possess a high industrial potential as the most common breeding species on specialized farms—helicultures [86].

The aim of this paper is to use, for the first time, 3D chitin scaffolds isolated from the giant verongiid demosponge *A. archeri* (Figure 1) for molluscs hemolymph-associated biomineralization ex vivo. This experimental study was additionally motivated by the fact that both amorphous and crystalline phases (i.e., calcite) of calcium carbonates are known to be bio-compatible [87,88,89], biodegradable [90,91,92] and osteoinductive substrates for tissue engineering of bones and other hard tissues [93,94,95,96,97,98,99,100,101,102,103,104,105].

## 2. Results

**Figure 3:**
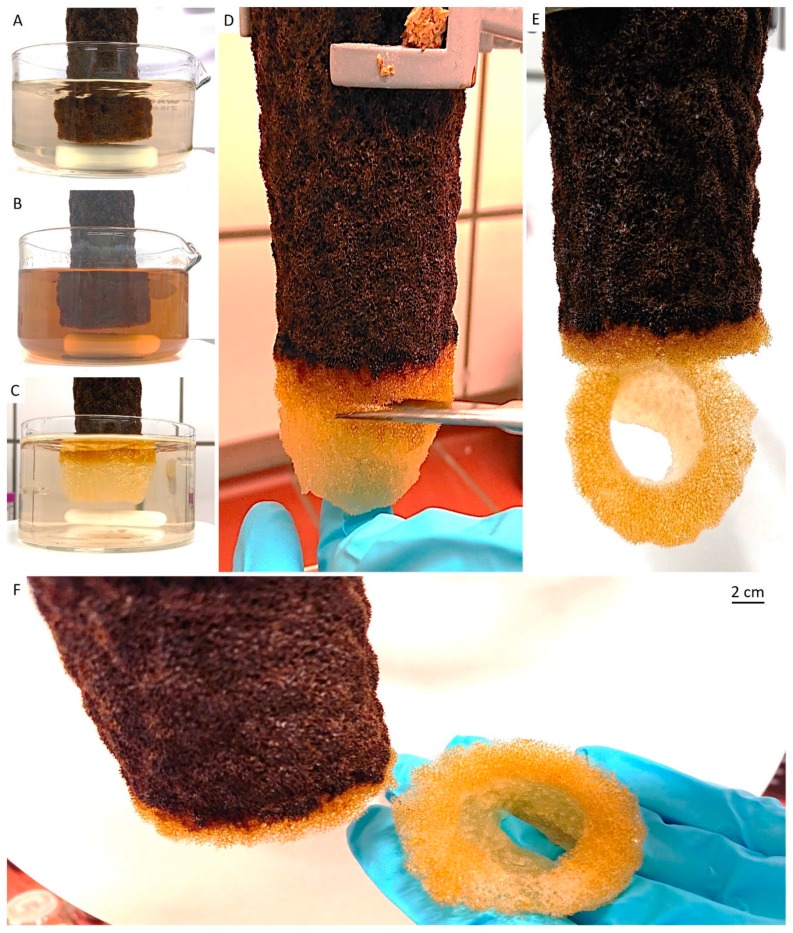
Step-by-step isolation of selected fragment of 3D tubular chitin scaffold from dried *A. archeri* segment. In step I, water-soluble salts were removed by pretreatment of the sponge skeleton with distilled water in the beaker placed on a magnetic stirrer (**A**) for 6 h. Afterward, in step II, the sponge was treated with 2.5 M of NaOH solution during 24 h, for deproteinization and depigmentation (**B**). In step III, the 3D scaffolds were treated with 20% acetic acid for 24 h, to remove residual calcium and magnesium carbonates, followed by washing with distilled water up to pH 6.8 (**C**). Steps II and III were repeated twice to obtain tubular scaffolds (**D**,**E**), which were cut off from the sponge and used for further studies (**F**).

**Figure 4:**
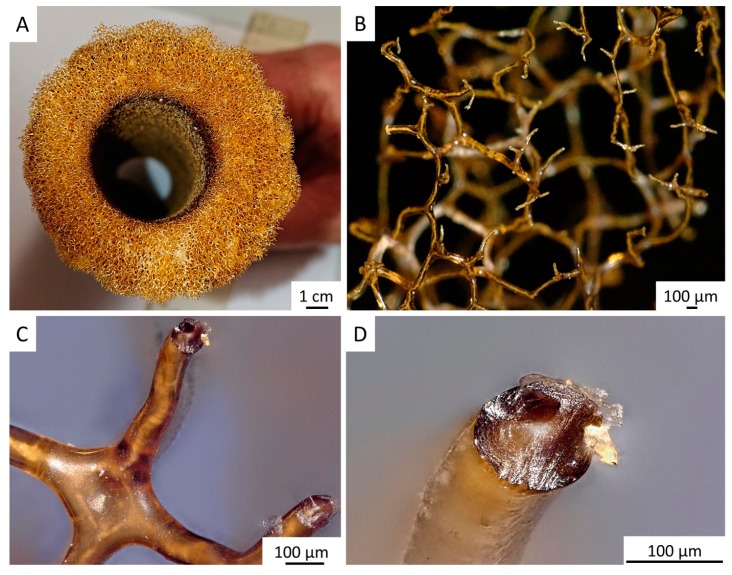
Decellularized chitinous skeleton of selected fragment of *A. archeri* (**A**) possesses anatomizing 3D microporous architecture (**B**) due to the presence of naturally mineralized, rigid fibers (**C**,**D**). The microstructure of skeletal fibers was characterized by us previously [4].

The tube-like body (Figure 1) of the demosponge *A. archeri* represents a unique, naturally predesigned, hierarchically structured fibrous construct (Figure 3 and Figure 4). Such tubular constructs can be up to 150 cm long, with an inner diameter of up to 10 cm (for morphological details, see [106,107,108,109]). The color of the live sponge ranges from lavender through gray to brown; however, air-dried sponges turn reddish-brown (Figure 3) (see also [4]). Recently, we unambiguously showed the chitinous origin of *A. archeri* skeletal tubes [4]. The chitin content of *A. archeri* skeleton reaches ~5% by weight [4]. Consequently, now, we take the liberty of suggesting that such chitinous constructs represent the longest tubular chitin known. This unique source of pre-designed microporous chitin has a distinct advantage for practical applications: researchers, according to their aims, can use selected fragments (Figure 4) of specific size from the hard skeletal tube. For example, the long fragment of the tube can be fixed (Figure 3D) so that only the lower part is the subject for treatment (Figure 3A–C) with respect to the isolation of pure chitin scaffold (Figure 3F and Figure 6).

**Figure 5:**
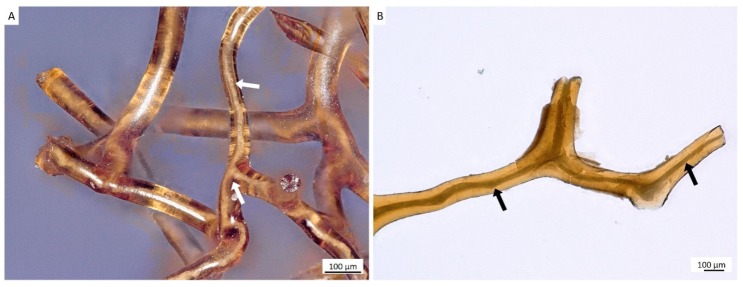
Alternating treatment of skeletal fibers of *A. archeri*, using alkali and acid solutions, leads to the visualization of inner channels (arrows) located within fibers (**A**,**B**). Such channels are responsible for capillary activity of these constructs with respect to water and other liquids. For details, see [4].

**Figure 6:**
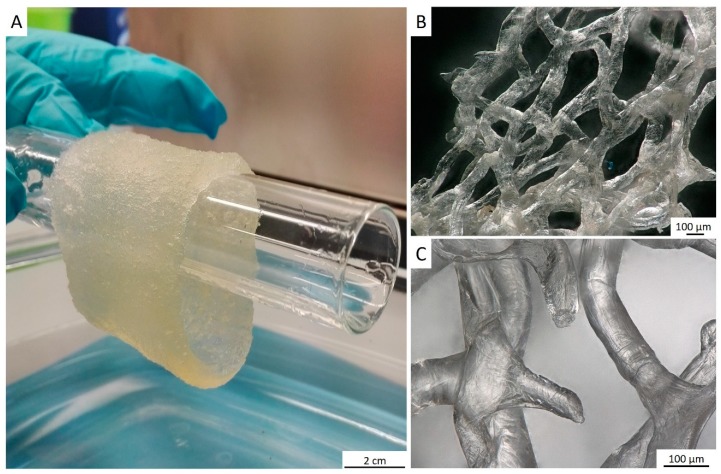
After purification, a translucent tube-like chitinous scaffold derived from *A. archeri* (see Figure 3) remains stable enough, but flexible after being saturated with water (**A**). It takes over the shape of respective hard surface, for example, this glass tube. The 3D architecture of the scaffold made of interconnected microtubes is very visible, using digital stereo microscopy (**B**,**C**). The porosity of such scaffold ranges between 300 and 800 µm.

For the isolation of ready-to-use chitinous scaffold (Figure 6), we used the standard alternating alkaline-acid-based treatment [24], which took in this case up to four days at room temperature. During this procedure, the presence of interconnected inner channels located within skeletal microtubes could be well-visualized (Figure 5). Following this simple treatment, the tube-like skeleton of *A. archeri* was completely demineralized, cell- and pigment-free, mechanically flexible (Figure 6A) and transparent.

Recently, we reported [4] that 3D microtubular chitin of *A. archeri* sponge can function as an effective capillary system with respect to model liquids such as methylene blue dye, crude oil and pig blood. Consequently, there are all the necessary preconditions to suggest that 3D chitinous scaffold isolated in this study can also function as liquid delivery construct [28] for the cell-growth medium, which could be, for example, optimized for the cultivation of hemocytes of mollusks origin.

**Figure 7:**
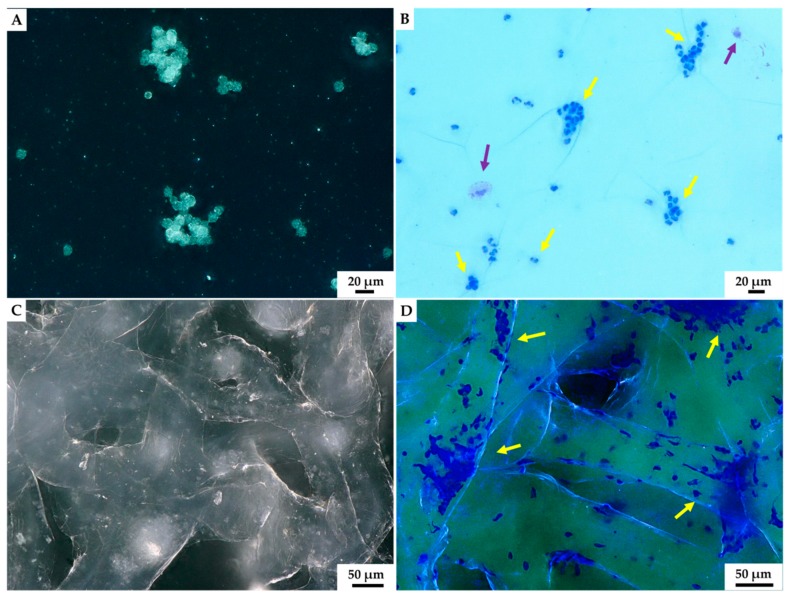
Light microscopy imagery. Characteristic aggregates of hemocytes present in isolated hemolymph of *C. aspersum* snail was observed both without (**A**) and using eosin and methylene blue staining (**B**) on the glass slide. The formation of hemocytes-based clumps before (**C**) and after staining by eosin and methylene blue (**D**) became visible on the surface of *A. archeri* chitinous scaffold 24 h after the immersion in hemolymph. Two hemocytes types could be distinguished after staining: most dominant granulocytes (yellow arrow) and single hyalinocytes (violet arrow).

**Figure 8:**
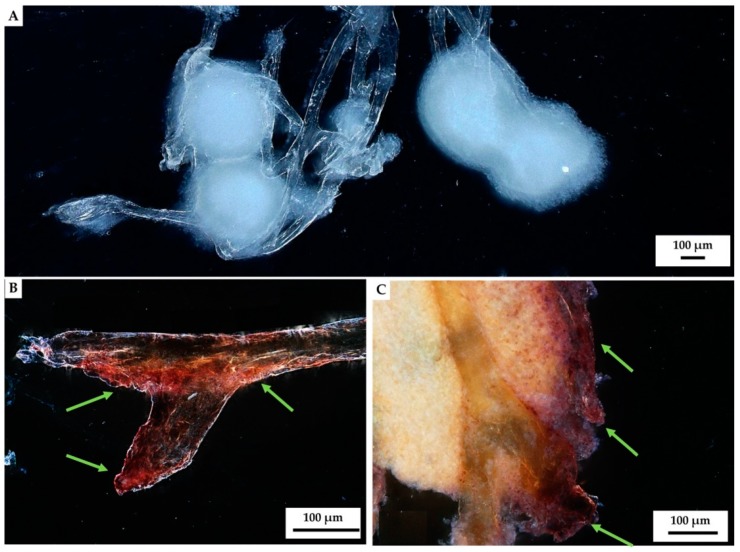
Stereo microscopy imagery of hemocyte clusters formed on the fibers of *A. archeri* chitinous scaffolds. Round white-colored clusters (**A**) of disintegrating hemocytes appeared 48 h after chitin immersion into the hemolymph isolated from *C. aspersum* snail (Figure 2 and Figure 14). Calcium-rich cytoplasmic dense microparticles (arrows) within the disintegrating granulocytes became visible after staining with Alizarin Red S on the chitinous fiber (**B**), as well as on the surface of the hemocytes cluster (**C**).

Herein, for the first time, we show how short-term cultivated *C. aspersum* hemocytes interact with the 3D chitinous scaffolds isolated from marine sponge *A. archeri* (Figure 7). According to Yoshino and co-authors [110], hemocytes cultured in vitro, without any additional culture medium, are able to survive for two to three days, and this was further confirmed by our results. The presence of hemocytes in the hemolymph of *C. aspersum* snail was shown by using eosin and methylene blue staining, according to the protocol of Grandiosa et al. [111]. At 24 h following the contact of hemolymph with sponge chitin, the formation of hemocytes clusters (see Figure 7C,D) at chitinous scaffolds became quite visible. Then, 48 h after the contact, hemocytes clusters enlarged in size (see Figure 8).

Our results indicated that hemocytes attached to the surface of external chitinous matrix. Most of the distinguished hemocytes were represented by granulocytes visible in light microscopy images (Figure 7B,D). Aggregation of hemocytes is natural and has been observed in 27 preparations of chitinous scaffolds immersed in hemolymph. Hemocytes are capable of ameboid movement, and they demonstrate the formation of nodules and positive chemotactic behavior in response to foreign substances. The appearance of long pseudopodia branching on the surface of tested chitinous material was visualized (Figure 7). Alizarin Red S staining confirmed the presence of calcium-rich deposits located on the chitin fibers (green arrow, Figure 8B,C), as well as on the cluster of disintegrated granulocytes, after 48 h (see Figure 8). It is possible that both the immune response of hemocytes and the disintegrations of their calcium-rich cytoplasm are crucial for biomineralization *ex vivo*.

**Figure 9:**
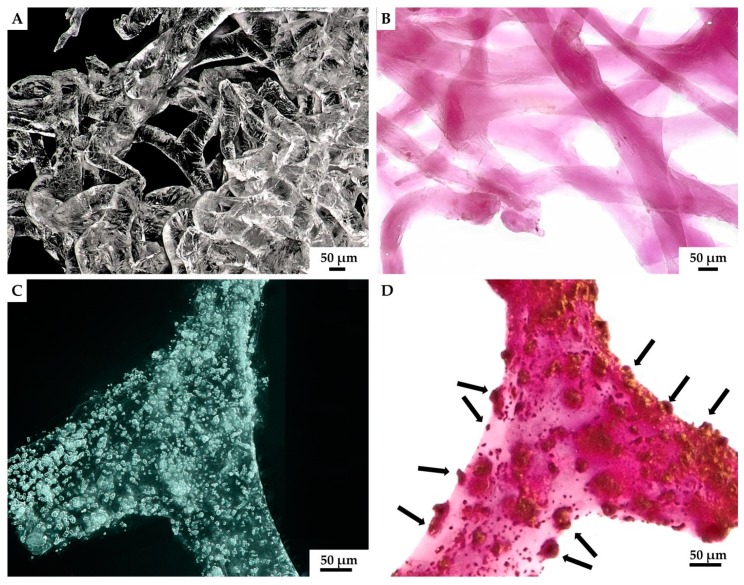
Isolated 3D chitinous scaffolds from *A. archeri* prior to ex vivo biomineralization with *C. aspersum* hemolymph (**A**) became slightly violet after Alizarin Red S staining (**B**). However, the formation of granular calcium-based deposits on chitin surface (**C**) after biomineralization ex vivo is quite visible when using digital light microscopy due to the intensive red coloration (**D**) with the same stain.

Thus, the formation of calcium-based phase on the surface of chitinous matrix after its immersion into hemolymph of *C. aspersum* snail can be easily confirmed by using Alizarin Red S staining (Figure 9). However, this raises an intriguing question regarding the nature of these calcium-containing granular deposits, which theoretically contain calcite, aragonite and vaterite, as well as amorphous calcium carbonate, according to previous reports [82,112,113].

**Figure 10:**
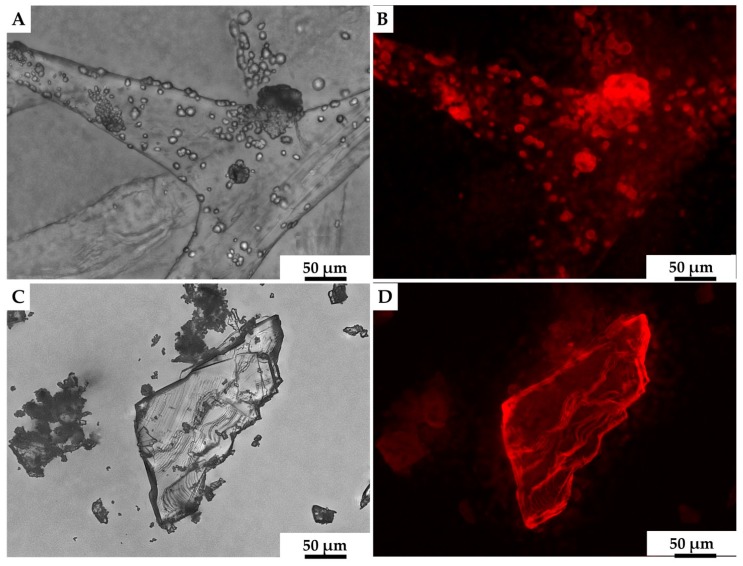
Both calcite mineral standard (**A**) and the mineral phase observed after ex vivo biomineralization of 3D chitin scaffold, using hemolymph of *C. aspersum* snail (**C**), possess very similar features with respect to their auto-fluorescence [116] (**B**,**D**, respectively).

**Figure 11:**
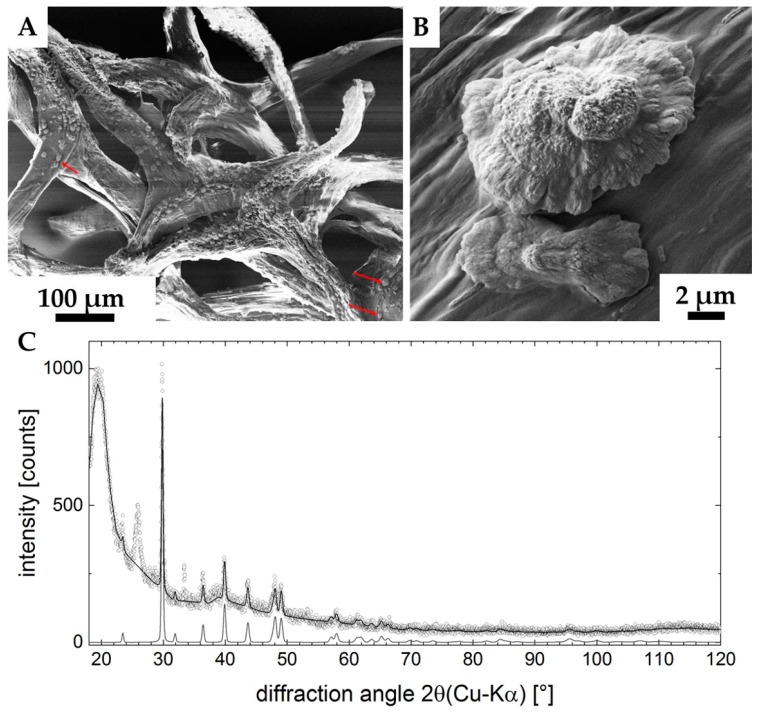
SEM images (**A**,**B**) of the ex vivo mineralized surface of 3D chitinous scaffold confirm the presence of microcrystallites (arrows). X-ray diffraction pattern of the sample is represented in the image **C**. Measured data are open dots; the refinement is highlighted by the solid line. The diffractogram of calcite-CaCO_3_ is shown at the bottom, clearly verifying the presence of this phase in the sample. Two unassigned peaks remain in the data (2θ ≈ 27°, 2θ≈ 33°). The large feature at 2θ ≈ 20° indicates a high content of amorphous material in the sample (**C**).

**Figure 12:**
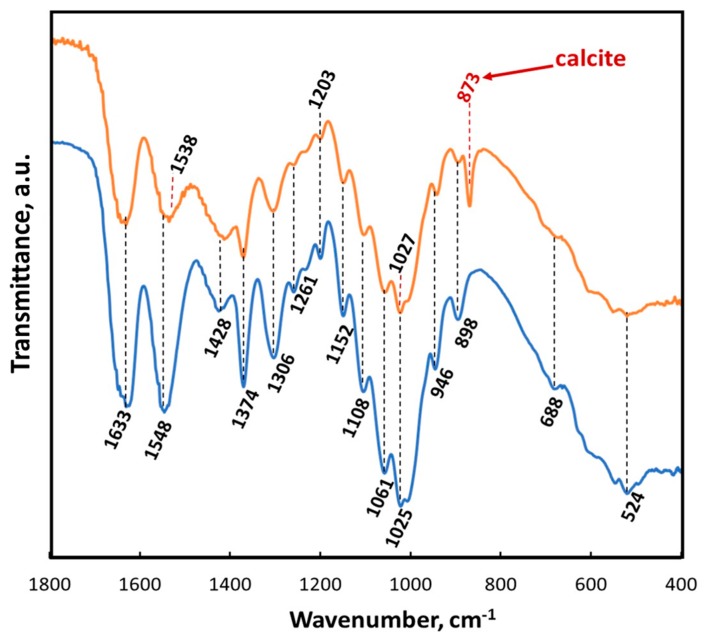
ATR-FTIR spectra of *A. archeri* chitin scaffold before (blue line) and after ex vivo biomineralization (orange line) within the region of 1800–400 cm^−1^.

**Figure 13:**
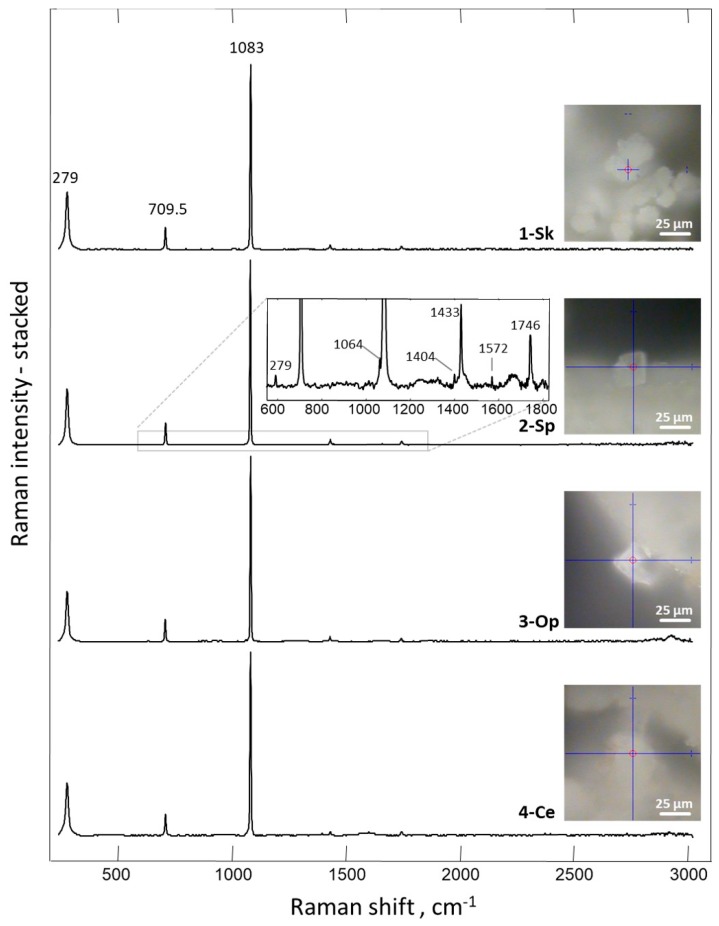
Raman spectrum of the mineral phase obtained on the surface of 3D chitinous scaffold after ex vivo biomineralization mediated by snail hemolymph.

We assumed that calcite was likely to be the biomineral formed ex vivo by hemocytes on the surface of 3D chitinous scaffolds (Figure 9C,D), and so we carried out the initial analysis of deposits, using fluorescence microscopy. The results obtained (Figure 10) clearly show a high similarity of red auto-fluorescence between calcite standard [114,115] and microgranular deposits formed after biomineralization ex vivo, using *C. aspersum* hemolymph. To analyze the obtained biomineral with respect to calcite composition, we used traditional analytical techniques, such as X-ray diffraction analysis (XRD) (Figure 11), FTIR (Figure 12) and Raman (Figure 13) spectroscopy.

The X-ray diffraction pattern of the sample is represented in Figure 11 C. It shows the characteristics of a sample with a high amorphous content (big hump at 2θ ≈ 20°). Superimposed, a set of peaks being characteristic for Calcite-CaCO_3_ (ICDD #04-012−0489) were found. The pattern still shows two unidentified peaks (2θ ≈ 27°, 2θ ≈ 33°), none of which provides evidence for the presence of a second CaCO_3_ modification (Aragonite, Vaterite or the structures containing crystal water). According to the database search, these two peaks may originate from a crystallized organic component in the system. XRD analysis (Rietveld refinement) pointed to the differences between the measured lattice parameters and the database entry. The refined lattice parameters are a = (4.973 ± 0.001) Å (database: a = 4.987 Å) and c = (16.987 ± 0.001) Å (database: c = 17.058 Å), corresponding to a shrunk lattice.

The results of ATR-FTIR analysis of the chitinous scaffold used in this study before and after ex vivo biomineralization are represented in Figure 12. Both spectra show a characteristic split for α-chitin, such as amide I at 1633 cm^−1^ (see orange and blue lines). This band corresponds to the presence of stretching vibrations from intermolecular (C=O⋯HN) and intramolecular (C=O⋯HO(C6); C=O⋯HN) hydrogen bonds [59,117]. The observation of such bands as amide II (νN–H and νC–N) at 1548 cm^−1^, amide III (νC–N and δN–H) at 1306 cm^−1^ or characteristic intense band at 898 cm^−1^ (C–O–C bridge as well as β-glycosidic linkage) additionally confirmed the presence of α-chitin [24]. A sharp peak visible at 873 cm^−1^ (red arrow) clearly indicated that calcium carbonate as calcite polymorph (monohydrocalcite) [117,118,119,120] appeared on the chitinous scaffold after ex vivo biomineralization using hemolymph isolated from *C. aspersum* (see Figure 12).

The results obtained by employing Raman spectroscopy clearly showed (Figure 13) that the spectrum recorded for crystals obtained after ex vivo biomineralization corresponds to the spectra acquired for the biogenic calcite [121,122]. The most intensive band, with the maximum near 1083 cm^−1^, corresponds to ν1 symmetric stretching vibrations CO_3_^2−^ of calcite [121,123], where the peak detected at 1433 cm^−1^ is related to CO_3_^2−^ ν_2_ asymmetric stretching vibrations [124]. Additional evidence of calcite polymorph presence is provided by the bands at 711 and 279 cm^−1^. These two peaks are related to ν4 in-plane bending vibrations of CO_3_^2−^ and vibrational modes of calcite, respectively [124]. Moreover, all present bands are consistent with calcite, as reported for the natural mineral in the RRUFF database (http://www.rruff.info/ (03.12.2019))

## 3. Discussion

It is recognized that “the construction of novel polymer materials via physical approaches and green technology from raw chitin is a sustainable pathway and beneficial to the green development” [58]. However, this approach is connected with numerous preparative steps, including, for example, mechanochemical disassembly, particulate leaching, gas foaming, thermally induced phase separation, electrospinning, dissolution of chitin in special liquids [122,125,126], and its supercritical drying [127]. Alternatively, diverse fractions of flakes, powders and nano forms of chitin (i.e., nanowhiskers, nanofibrils and nanocrystals) [128,129,130] can be obtained from demineralized and deproteinated crustaceans’ chitin. Such scaffolding strategies [131], however, are connected with technological difficulties and other disadvantages which can be a critical weakness in terms of cost and future clinical use [132].

As mentioned in the introduction, marine verongiid sponges produce practically ready-to-use 3D constructs made of microtubular, mechanically robust, yet flexible scaffolds, which are very well applicable for the cultivation of diverse human cells. However, would they be equally as good scaffolds for hemocytes of invertebrate origin?

The hemolymph of mollusks, including terrestrial snails, is mostly composed of water and contains hemocyanins, diverse inorganic ions, metabolites, lectins and enzymes, as well as the number of cells (i.e., blastocytes, hyalinocytes and granulocytes) [133,134,135,136] known as hemocytes. Granulocytes, making up over 95% of circulating hemocytes, are 9–50 µm in size, have an irregular nucleus and cytoplasm containing numerous granules [137]. On a flat surface, they exhibit ameboid motion and are able to form visible pseudopodia. These cells are capable of phagocytosis and its more complex forms, requiring collective participation of many granulocytes: encapsulation and nodulation [113]. Furthermore, granulocytes of the marine mollusk *Pinctada fucata* were reported to contain numerous calcium-rich vesicles and crystals serving as calcium pool [113].

Molluscan in vitro cell culture systems have a huge impact on our understanding of their complex physiological processes, functions and relation between tissue-specific cells [110]. Our study represents the first data concerning the ability of *C. aspersum* hemocytes to grow on chitinous matrices principally (Figure 7) and, furthermore, to build clusters (Figure 8). These phenomena may be explained as follows. Firstly, hemocytes are cells responsible for immune responses [138,139,140,141] recognizing sponge *a*-chitin as a foreign body. Nodulation in terrestrial snails is a multi-stage process, requiring the presence of many granulocytes. This process is initiated by the contact of the granulocyte with a foreign substance. As a result of degranulation occurring in the granular cytoplasm, chemotaxis-related substances are released. They, in turn, attract other hemocytes to the site where the foreign matter is encapsulated by many layers of granulocytes (Figure 7). Moreover, this site becomes a condensation center of calcium ions present in hemolymph, as well as the ones released from cytoplasm of hemocytes.

A second explanation could be related to biomineralization. Previously, chitin within mollusk shells has been detected, next to proteins, being a part of organic membrane, which appears during shell repair or formation [81,142,143]. It is possible that hemocytes identify chitinous matrix as a substituent of this membrane. In the literature, we can find undeniable evidence concerning active participation of hemocytes in calcification in vivo [80,81,82,144,145]. For example, Kapur and Sen Gupta (1970) [146] described such a membrane as a specialized scaffold for the granulocytes, which lose their identity, leaving free nuclei and organic matter, and deposit their calcium-rich granules from cytoplasm. In this case, the organic membrane acts as a crystal’s nucleation center. Similar explanations are to be found in numerous papers by Abolins-Krogis, who conducted studies on terrestrial snails from the *Helix* genus [79,144,147,148].

Biomimetic materials displaying the complexity of biominerals are artificially difficult to prepare [149], even though numerous in vitro attempts have been carried out previously [150,151,152,153,154,155,156]. Our results are in a good agreement with recently published data demonstrating that chitin–CaCO_3_ and porous chitin–CaCO_3_ scaffolds facilitated hMSCs attachment following cell osteogenic differentiation. However, such chitin scaffolds were prepared by dissolving crustaceans chitin flakes in LiCl/dimethylacetamide, following by gel formation and freeze-drying [157]. In contrast, the experimental results reported in this study unambiguously show a new biomimetic direction to apply already naturally prefabricated [18] 3D chitinous scaffolds of poriferan origin as templates for both the growth of mollusk and hemocytes and the functionalization of the surface of chitin fibers with respect to the formation of amorphous and crystalline (calcite) calcium carbonate-based layers. Our approach is based on ready-to-use 3D scaffolds of sponges’ origin hence only the size of already naturally predesigned pores could be a limiting factor. However, this can be neglected due to the ability of hemocytes not only to attach to the surface of individual chitin fibers, but also to build large-size clusters located between fibers that possess biomineralization activity (Figure 8).

Thus, our study resulted in the development of a new biomimetic product, utilizing mollusk hemocytes, which is based on ex vivo synthetized ACC and calcite tightly bound to the surface of sponge chitin used as 3D scaffold. The outlook of this biomimetic direction includes numerous open questions concerning the role of hemocytes in the creation of a fine-tuned microenvironment [158] which is necessary for calcification ex vivo. Without doubt, the mechanisms and kinetics of this kind of biomineralization should be investigated, in addition to carrying out further studies on mechanical properties of developed mineralized scaffolds aimed at practical application in biomedicine, in the near future.

## 4. Materials and Methods

### 4.1. Sample Location and Collection

#### 4.1.1. Aplysina archeri Sponges

The Caribbean sponges were collected and preserved (in 96% ethanol) as hosts of symbiotic invertebrates in the Bay of Pigs of Cuba (coordinates: 22°07’15.9″N, 81°07’07.2″W; depth: 21 m; date: 05 February 2019) and Playa Lagun of Curaçao (coordinates: 12°19’02″N, 69°09’09″W; depth: 20 m; date: 20 June 2017). Stove-pipe sponge *Aplysina archeri* (Higgin, 1875) (previously known as *Luffaria archeri* or *Verongia archeri* (see Van Soest et al., 2019) (phylum Porifera, class Demospongiae, order Verongiida, family Aplysinidae).

#### 4.1.2. *Cornu aspersum* Snails

One-year-old snails, *Cornu aspersum*, were obtained from a commercial heliculture farm from the central part of Poland (Snails Breeder—Hodowla Ślimaków, Małszyce, Poland). Snails were maintained in a glass aquarium, at room temperature. As food, lettuce, carrots and apples were used. Moreover, cuttlebone of *Sepia officinalis* cuttlefish was given to snails as calcium carbonate enrichment (CaCO_3_) in the diet. The snails were kept moist throughout the experimental period with wet humus. Animal rights statement is not required.

### 4.2. Isolation Procedure

#### 4.2.1. Isolation of Chitinous Scaffolds from the *A. archeri* Demosponge Skeleton

The procedure for the isolation of chitin-based scaffolds from verongiid sponges, as reported by us [24], was followed here. In brief, it was performed accordingly to the following steps: (I) sponge skeleton was washed three times with distilled water in the vessel on a magnetic stirrer, to remove water-soluble compounds, for 6 h (Figure 3A). Afterward, the water was changed (step II) with 2.5 M of NaOH, at 37 °C, for 24 h, for deproteinization and depigmentation (Figure 3B). This step was then followed by (step III) treatment with 20% CH_3_COOH at 37 °C, for a period of 24 h, to remove residual calcium and magnesium carbonates and then washing in distilled water up to pH 6.8. Steps II and III were repeated two times, to obtain colorless tubular scaffolds (Figure 3D–F). The purity of isolated chitin scaffolds has been proved according to standard analytical procedures as described previously [4].

#### 4.2.2. Nonlethal Isolation of the Hemolymph from the *C. aspersum* Snail

**Figure 14:**
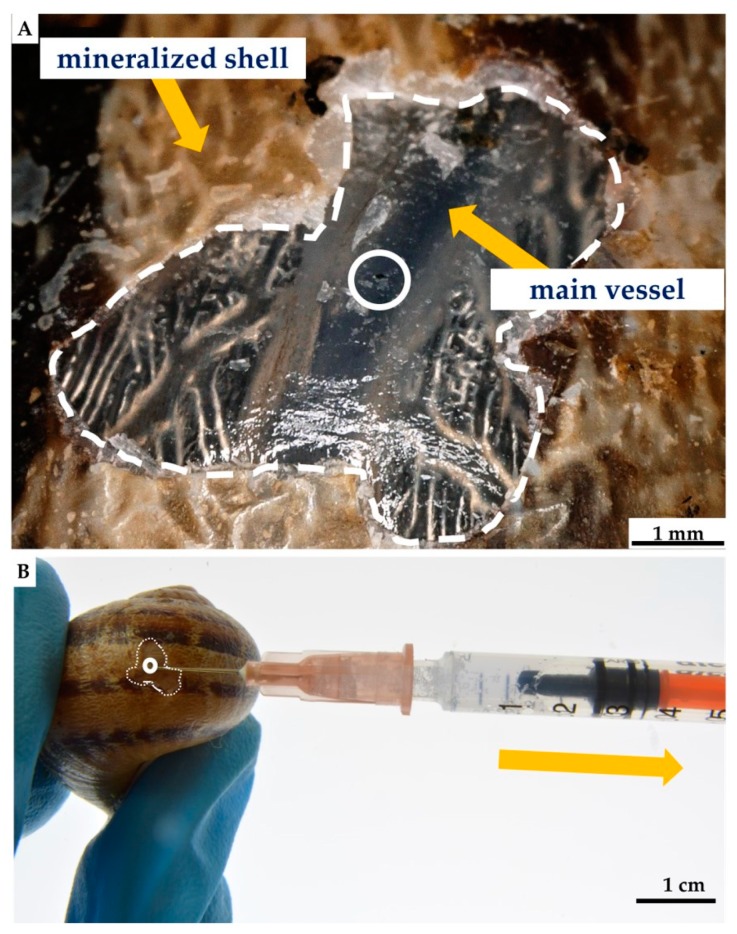
Nonlethal extraction of the hemolymph from cultivated *C. aspersum* snail, using syringe (**A**). Initially, the shell of the snail was disrupted mechanically by using a scalpel, with the aim of obtaining around 5 mm × 5 mm large shell-free surface. After this procedure, the location of the main “blood” vessel becomes visible (**B**) and accessible to use a needle for the hemolymph extraction (**A**).

For hemolymph collection, the modified nonlethal intravital method described in Polish patent P.410296 and article [159] was used. The shell surface of the selected *C. aspersum* snail was cleaned with 70% ethanol. After that, a piece of shell was removed, and 3 h later, about 1000 μL of hemolymph was isolated from an individual of *C. aspersum* by main-vessel puncture, using a sterile syringe and needle [159] (see Figure 14). Twelve hours post-isolation, an organic film was observed in the site of removed shell fragment, which was fully mineralized within the next few days [160]. During the next three months after the procedure, the snails used in this study did not show any visible changes in their physiology or in behavior.

### 4.3. Ex Vivo Biomineralization of the A. archeri Chitinous Scaffolds

A square fragment (10 × 10 mm) of selected chitinous scaffold (Figure 10) was immersed in hemolymph of *C. aspersum* for one hour and then placed on a sterile slide, until completely dried, at room temperature. The procedure was replicated five times. The described technique aimed to simulate biomineralization of organic matrix by snail hemolymph under natural conditions via mimicking of physicochemical effects reported for shell regeneration of terrestrial snails previously [161,162]. Obtained in this way, mineral phases deposited on chitinous matrix were characterized, as represented below.

### 4.4. Short-Term Cultivation of Hemocytes on Chitinous Matrix

For short-term cultivation of hemocytes, about 0.5 mL of the hemolymph was isolated, using the method described above, and placed in a 2 mL Eppendorf tube. Selected fragment of chitinous matrix was immersed in the hemolymph, at room temperature, for 24 and 48 h, respectively. To prevent possible contamination with bacteria streptomycin (100 μg/mL) and penicillin (60 μg/mL) have been used [163].

### 4.5. Characterization of Obtained Materials

#### 4.5.1. Photography and Figures

Photographs and macroscopic images were performed by Nikon D-7100 camera with Nikon AF-S DX 18–105 mm f/3.5-5.6G and Nikon AF-S VR Micro-Nikkor 105 mm f/2.8G IF-ED objective lenses. Figures were prepared, using the GNU Image Manipulation Program GIMP and Microsoft Office tool PowerPoint 2016.

#### 4.5.2. Digital, Light and Fluorescence Microscopy

The obtained samples were observed, using an advanced imaging and measurement system consisting of Keyence VHX-6000 digital optical microscope and the swing-head zoom lenses VH-Z20R (magnification up to 200×) and VH-Z100UR (magnification up to 1000×). The light and fluorescence microscope modes were performed with a BZ-9000 (Keyence) microscope.

#### 4.5.3. Eosin and Methylene Blue Staining

We used Hemavet (Kolchem) as the combination of eosin and methyl blue dyes. Previously, this stain was successfully used for hemocytes detection and characterization [111,164,165]. Hemolymph cell monolayers (HCMs) were prepared by spreading the drop of hemolymph on sterile glass slide and drying it at ambient temperature. Staining was also used to detect hemocytes settled on chitinous scaffold (see Figure 7).

#### 4.5.4. Alizarin Red S Staining

Ex vivo mineralized chitinous scaffolds were stained with Alizarin Red S (Sigma-Aldrich) and compared with that of native *A. archeri* chitin as isolated. For the staining procedure, 40 mM of Alizarin Red S (pH 8.3) was used for staining of the samples, during 30 min, at room temperature (for details see [165]). Stained samples were washed with distilled water five times, to eliminate the unattached Alizarin Red S, as well as mineral particles which were not tightly attached to the surface of chitin fibers. Calcium deposits were detected as orange–red color particles, using digital microscopy.

#### 4.5.5. Fluorescent Microscopy Analysis

Materials in this study were observed with a BZ-9000 fluorescence microscope (Keyence, Osaka, Japan), in fluorescent and light microscopy mode. Calcite mineral standard was purchased from the International Institute of Biomineralogy (INTIB GmbH), Freiberg, Germany.

#### 4.5.6. ATR FT-IR and Raman Spectroscopy

Infrared spectroscopy techniques were used for the qualitative characterization of obtained mineralized scaffolds, as well as pure chitin isolated from *A. archeri*. The presence of expected functional group was confirmed by ATR FT-IR (attenuated total reflectance Fourier transform infrared spectroscopy) and verified, using Nicolet 210c spectrometer (Thermo Scientific). The investigation was performed over a wavenumber range of 1800–400 cm^−1^ (resolution of 0.5 cm^−1^). Raman spectra were recorded, using a Raman spectrometer (Raman Rxn1TM, Kaiser Optical Systems Inc., Ann Arbor, USA) coupled to a light microscope (DM2500 P, Leica Microsystems GmbH, Wetzlar, Germany).

#### 4.5.7. XRD

The phases in the sample were identified through X-ray diffraction, using a SEIFERT-FPM URD6 diffractometer equipped with a sealed X-ray tube with Cu anode. The device operated in symmetrical Bragg–Brentano geometry with a secondary graphite monochromator in front of the proportional counter detector. For the measurement, the sample was fixed with Scotch Tape to a ‘zero background’ to a sample holder. Phase identification was performed with the ICDD PDF-4+ database linked to the Panalytical HighScore+ software. Rietveld refinement for a more detailed analysis of the sample was conducted with the Maud software package.

#### 4.5.8. Scanning Electron Microscopy (SEM)

Scanning electron microscopy was performed using XL 30 ESEM Philips-Scanning Electron Microscope (FEI Company, Peabody, MA, USA). Samples were fixed on a sample holder with carbon patches and then covered with carbon or with a 5–10 nm gold layer using Edwards Sputter Coater S150B (BOC Edwards, Wilmington, MA, USA).

## References

[1] Su X., Tan M., Duan B., Cai J., Jiang W., Zhang L. (2019). Hierarchical microspheres with macropores fabricated from chitin as 3D cell culture. J. Mater. Chem. B.

[2] Morganti P., Coltelli M.B., Santi S. (2018). Biobased tissues for innovative cosmetic products: Polybioskin as an EU research project. Glob. J. Nanomedicine.

[3] Loh Q.L., Choong C. (2013). Three-dimensional scaffolds for tissue engineering applications: role of porosity and pore size. Tissue Eng. Part B Rev..

[4] Klinger C., Żółtowska-Aksamitowska S., Wysokowski M., Tsurkan M.V., Galli R., Petrenko I., Machałowski T., Ereskovsky A., Martinović R., Muzychka L. (2019). Express Method for Isolation of Ready-to-Use 3D Chitin Scaffolds from *Aplysina archeri* (Aplysineidae: Verongiida) Demosponge. Mar. Drugs.

[5] Zhang X., Vecchio K.S. (2013). Conversion of natural marine skeletons as scaffolds for bone tissue engineering. Front. Mater. Sci..

[6] Silva T.H., Moreira-Silva J., Marques A.L.P., Domingues A., Bayon Y., Reis R.L. (2014). Marine origin collagens and its potential applications. Mar. Drugs.

[7] Nisticò R. (2017). Aquatic-Derived Biomaterials for a Sustainable Future: A European Opportunity. Resources.

[8] Teimouri A., Azadi M., Shams Ghahfarokhi Z., Razavizadeh R. (2017). Preparation and characterization of novel β-chitin/nanodiopside/nanohydroxyapatite composite scaffolds for tissue engineering applications. J. Biomater. Sci. Polym. Ed..

[9] Neto A.S., Ferreira J.M.F. (2018). Synthetic and marine-derived porous scaffolds for bone tissue engineering. Materials.

[10] Lim Y.S., Ok Y.J., Hwang S.Y., Kwak J.Y., Yoon S. (2019). Marine collagen as a promising biomaterial for biomedical applications. Mar. Drugs.

[11] Yadav M., Goswami P., Paritosh K., Kumar M., Pareek N., Vivekanand V. (2019). Seafood waste: a source for preparation of commercially employable chitin/chitosan materials. Bioresour. Bioprocess..

[12] Green D.W., Lai W.F., Jung H.S. (2014). Evolving marine biomimetics for regenerative dentistry. Mar. Drugs.

[13] Choi A.H., Ben-Nissan B. (2019). Marine-Derived Biomaterials for Tissue Engineering Applications.

[14] Wysokowski M., Motylenko M., Beyer J., Makarova A., Stöcker H., Walter J., Galli R., Kaiser S., Vyalikh D., Bazhenov V.V. (2015). Extreme biomimetic approach for developing novel chitin-GeO_2_ nanocomposites with photoluminescent properties. Nano Res..

[15] Zdarta J., Antecka K., Frankowski R., Zgoła-Grześkowiak A., Ehrlich H., Jesionowski T. (2018). The effect of operational parameters on the biodegradation of bisphenols by *Trametes versicolor* laccase immobilized on *Hippospongia communis* spongin scaffolds. Sci. Total Environ..

[16] Petrenko I., Summers A.P., Simon P., Zółtowska-Aksamitowska S., Motylenko M., Schimpf C., Rafaja D., Roth F., Kummer K., Brendler E. (2019). Extreme biomimetics: Preservation of molecular detail in centimeter-scale samples of biological meshes laid down by sponges. Sci. Adv..

[17] Kovalchuk V., Voronkina A., Binnewerg B., Schubert M., Muzychka L., Wysokowski M., Tsurkan M.V., Bechmann N., Petrenko I., Fursov A. (2019). Naturally drug-loaded chitin: isolation and applications. Mar. Drugs.

[18] Schubert M., Binnewerg B., Voronkina A., Muzychka L., Wysokowski M., Petrenko I., Kovalchuk V., Tsurkan M., Martinovic R., Bechmann N. (2019). Naturally prefabricated marine biomaterials: isolation and applications of flat chitinous 3D scaffolds from *Ianthella labyrinthus* (Demospongiae: Verongiida). Int. J. Mol. Sci..

[19] Ehrlich H. (2019). Marine Biological Materials of Invertebrate Origin.

[20] Green D., Howard D., Yang X., Kelly M., Oreffo R.O.C. (2003). Natural marine sponge fiber skeleton: A biomimetic scaffold for human osteoprogenitor cell attachment, growth, and differentiation. Tissue Eng..

[21] Green D.W. (2008). Tissue bionics: examples in biomimetic tissue engineering. Biomed. Mater..

[22] Jesionowski T., Norman M., Żółtowska-Aksamitowska S., Petrenko I., Joseph Y., Ehrlich H. (2018). Marine Spongin: Naturally Prefabricated 3D Scaffold-Based Biomaterial. Mar. Drugs.

[23] Ehrlich H., Shaala L.A., Youssef D.T.A., Żółtowska- Aksamitowska S., Tsurkan M., Galli R., Meissner H., Wysokowski M., Petrenko I., Tabachnick K.R. (2018). Discovery of chitin in skeletons of non-verongiid Red Sea demosponges. PLoS One.

[24] Ehrlich H., Ilan M., Maldonado M., Muricy G., Bavestrello G., Kljajic Z., Carballo J.L., Schiaparelli S., Ereskovsky A., Schupp P. (2010). Three-dimensional chitin-based scaffolds from Verongida sponges (Demospongiae: Porifera). Part I. Isolation and identification of chitin. Int. J. Biol. Macromol..

[25] Ehrlich H., Steck E., Ilan M., Maldonado M., Muricy G., Bavestrello G., Kljajic Z., Carballo J.L., Schiaparelli S., Ereskovsky A. (2010). Three-dimensional chitin-based scaffolds from Verongida sponges (Demospongiae: Porifera). Part II: Biomimetic potential and applications. Int. J. Biol. Macromol..

[26] Ehrlich H., Bazhenov V.V., Debitus C., de Voogd N., Galli R., Tsurkan M.V., Wysokowski M., Meissner H., Bulut E., Kaya M. (2017). Isolation and identification of chitin from heavy mineralized skeleton of *Suberea clavata* (Verongida: Demospongiae: Porifera) marine demosponge. Int. J. Biol. Macromol..

[27] Brunner E., Ehrlich H., Schupp P., Hedrich R., Hunoldt S., Kammer M., Machill S., Paasch S., Bazhenov V.V., Kurek D.V. (2009). Chitin-based scaffolds are an integral part of the skeleton of the marine demosponge *Ianthella basta*. J. Struct. Biol..

[28] Wysokowski M., Bazhenov V.V., Tsurkan M.V., Galli R., Stelling A.L., Stöcker H., Kaiser S., Niederschlag E., Gärtner G., Behm T. (2013). Isolation and identification of chitin in three-dimensional skeleton of *Aplysina fistularis* marine sponge. Int. J. Biol. Macromol..

[29] Żółtowska- Aksamitowska S., Tsurkan M.V., Lim S., Meissner H., Tabachnick K., Shaala L.A., Youssef D.T.A., Ivanenko V.N., Petrenko I., Wysokowski M. (2018). The demosponge *Pseudoceratina purpurea* as a new source of fibrous chitin. Int. J. Biol. Macromol..

[30] Żółtowska-Aksamitowska S., Shaala L., Youssef D., Elhady S., Tsurkan M., Petrenko I., Wysokowski M., Tabachnick K., Meissner H., Ivanenko V. (2018). First report on chitin in a non-Verongiid marine demosponge: the *Mycale euplectellioides* case. Mar. Drugs.

[31] Shaala L., Asfour H., Youssef D., Żółtowska-Aksamitowska S., Wysokowski M., Tsurkan M., Galli R., Meissner H., Petrenko I., Tabachnick K. (2019). New source of 3D chitin scaffolds: the Red Sea demosponge *Pseudoceratina arabica* (Pseudoceratinidae, Verongiida). Mar. Drugs.

[32] Fromont J., Żółtowska-Aksamitowska S., Galli R., Meissner H., Erpenbeck D., Vacelet J., Diaz C., Tsurkan M.V., Petrenko I., Youssef D.T.A. (2019). New family and genus of a Dendrilla-like sponge with characters of Verongiida. Part II. Discovery of chitin in the skeleton of *Ernstilla lacunosa*. Zool. Anz..

[33] Vacelet J., Erpenbeck D., Diaz C., Ehrlich H., Fromont J. (2019). New family and genus for Dendrilla-like sponges with characters of Verongiida. Part I redescription of *Dendrilla lacunosa Hentschel 1912*, diagnosis of the new family Ernstillidae and Ernstilla n. g. Zool. Anz..

[34] Binnewerg B., Schubert M., Voronkina A., Muzychka L., Wysokowski M., Petrenko I., Djurović M., Kovalchuk V., Tsurkan M., Martinovic R. (2020). Marine biomaterials: Biomimetic and pharmacological potential of cultivated *Aplysina aerophoba* marine demosponge. Mater. Sci. Eng. C.

[35] Anitha A., Sowmya S., Sudheesh K.P.T., Deepthi S., Chennazhi K.P., Ehrlich H., Tsurkan M., Jayakumar R. (2014). Chitin and chitosan in selected biomedical applications. Prog. Polym.Sci..

[36] Younes I., Rinaudo M. (2015). Chitin and chitosan preparation from marine sources. Structure, properties and applications. Mar. Drugs.

[37] Morganti P., Del Ciotto P., Carezzi F., Nunziata M.L., Morganti G. (2016). A chitin nanofibril-based non-woven tissue as medical dressing: The role of bionanotechnology. Nanomater Regen Med..

[38] Morganti P., Febo P., Cardillo M., Donnarumma G., Baroni A. (2017). Chitin nanofibril and nanolignin: Natural polymers of biomedical interest. J. Clin Cosmet Dermatol.

[39] Philibert T., Lee B.H., Fabien N. (2017). Current status and new perspectives on chitin and chitosan as functional biopolymers. Appl. Biochem. Biotechnol..

[40] Morganti P., Stoller M. (2017). Chitin and lignin: Natural Ingredients from waste materials for making innovative and healthy products for humans and plants. Chem. Eng. Trans..

[41] Morganti P., Del Ciotto P., Carezzi F., Morganti G., Hong-Duo C. (2012). From waste material a new anti-aging compound: a chitin nanofiber complex. SOFW J..

[42] Morganti P., Palombo M., Fabrizi G., Guarneri F., Svolacchia F., Cardillo A., Del Ciotto P., Francesco C., Gianluca M. (2013). New insight on anti-aging activity of chitin nanofibril-hyaluronan blocks copolymers entrapping active ingredients: In vitro and in vivo study. J. Appl. Cosmetol..

[43] Aranaz I., Acosta N., Civera C., Elorza B., Mingo J., Castro C., De los Llano Gandia M., Heras Caballer A. (2018). Cosmetics and cosmeceutical applications of chitin, chitosan and their derivatives. Polymers.

[44] Jayakumar R., Prabaharan M., Sudheesh Kumar P.T., Naira S.V., Tamura H. (2011). Biomaterials based on chitin and chitosan in wound dressing applications. Biotechnol. Adv..

[45] Mehrabani M.G., Karimian R., Rakhshaei R., Pakdel F., Eslami H., Fakhrzadeh V., Rahimi M., Salehi R., Kafil H.S. (2018). Chitin/silk fibroin/TiO_2_ bio-nanocomposite as a biocompatible wound dressing bandage with strong antimicrobial activity. Int. J. Biol. Macromol..

[46] Balassa L.L., Prudden J.F., Muzzarelli R.A.A., Pariser E.R. (1978). Applications of chitin and chitosan in wound-healing acceleration. Proceedings of the First International Conference on Chitin/Chitosan.

[47] Ohtsuki K., Ohnishi M., Nakamura Y., Kurokawa E. (1990). Clinical application of “Chitin”, artificial skin material in oral mucous defects. Jpn. J. Oral Surg..

[48] Kifune K. (1992). Clinical application of chitin artificial skin (Beschitin W). Advances in Chitin and Chitosan.

[49] Hirota Y., Tanioka S., Tanigawa T., Tanaka Y., Ojima R., Domard A., Jeuniaux C., Muzzarelli R., Roberts G. (1996). Clinical applications of chitin and chitosan to human decubitus. Advances in Chitin Science.

[50] Minami S., Okamoto Y., Shigemasa Y. (1998). Intensive skin activation in veterinary clinical application; how to utilize the natural resources chitin and chitosan *ZAIKAI Spec*. Issue.

[51] Gorovoj L., Seniouk O., Beketova G., Savichuk N., Amanbaeva G., Muzzarelli R.A.A. (2000). Use of the chitin-containing preparation Mycoton in pediatric gastroenterology. Chitosan per os: From Dietary Supplement to Drug Carrier.

[52] Fang Y., Hu Y., Wang Z., Zhou W., Yan L., Fan X., Liu H. (2020). 3D Porous Chitin Sponge with High Absorbency, Rapid Shape Recovery, and Excellent Antibacterial Activities for Noncompressible Wound. Chem. Eng. J..

[53] Ehrlich H., Ruys A.J. (2013). Biomimetic potential of chitin–based composite biomaterials of poriferan origin. Biomimetic Biomaterials: Structure and Applications.

[54] Su C.H., Sun C.S., Juan S.W., Hu C.H., Ke W.T., Sheu M.T. (1997). Fungal mycelia as the source of chitin and polysaccharides and their applications as skin substitutes. Biomaterials.

[55] Rahman M.A., Halfar J. (2014). First evidence of chitin in calcified coralline algae: New insights into the calcification process of *Clathromorphum compactum*. Sci. Rep..

[56] Connors M.J., Ehrlich H., Hog M., Godeffroy C., Araya S., Kallai I., Gazit D., Boyce M., Ortiz C. (2012). Three-dimensional structure of the shell plate assembly of the chiton *Tonicella marmorea* and its biomechanical consequences. J. Struct. Biol..

[57] Duan B., Huang Y., Lu A., Zhang L. (2018). Recent advances in chitin based materials constructed via physical methods. Prog. Polym. Sci..

[58] Merzendorfer H. (2011). The cellular basis of chitin synthesis in fungi and insects: Common principles and differences. Eur. J. Cell Biol..

[59] Kaya M., Mujtaba M., Ehrlich H., Salaberria A.M., Baran T., Amemiya C.T., Galli R., Akyuz L., Sargin I., Labidi J. (2017). On chemistry of γ-chitin. Carbohydr. Polym..

[60] Machałowski T., Wysokowski M., Żółtowska-Aksamitowska S., Bechmann N., Binnewerg B., Schubert M., Guan K., Bornstein S.R., Czaczyk K., Pokrovsky O. (2019). Spider Chitin. The biomimetic potential and applications of *Caribena versicolor* tubular chitin. Carbohydr. Polym..

[61] Machałowski T., Wysokowski M., Tsurkan M.V., Galli R., Schimpf C., Rafaja D., Brendler E., Viehweger C., Żółtowska-Aksamitowska S., Petrenko I. (2019). Spider chitin: an ultrafast microwave-assisted method for chitin isolation from *Caribena versicolor* spider molt cuticle. Molecules.

[62] Rohde S., Schupp P.J. (2011). Growth and regeneration of the elephant ear sponge *Ianthella basta* (Porifera). Ancient Animals, New Challenges.

[63] Verdenal B., Vacelet J., Rützler K. (1990). Sponge culture on vertical ropes in the northwestern Mediterranean Sea. New Perspect. Sponge Biology.

[64] Pronzato R., Bavestrello G., Cerrano C., Magnino G., Manconi R., Pantelis J., Sarà A., Sidri M. (1999). Sponge farming in the Mediterranean Sea: New perspectives. Mem. Queensl. Museum.

[65] Hausmann R., Vitello M.P., Leitermann F., Syldatk C. (2006). Advances in the production of sponge biomass *Aplysina aerophoba*- a model sponge for ex situ sponge biomass production. J. Biotechnol..

[66] Klöppel A., Pfannkunchen M., Putz A., Proksch P., Brümmer F. (2008). Ex situ cultivation of *Aplysina aerophoba* close to in situ conditions: ecological, biochemical and histological aspects. Mar. Ecol..

[67] Ehrlich H., Maldonado M., Parker A.R., Kulchin Y.N., Schilling J., Köhler B., Skrzypczak U., Simon P., Reiswig H.M., Tsurkan M.V. (2016). Supercontinuum generation in naturally occurring glass sponges spicules. Adv. Opt. Mater..

[68] Ehrlich H., Maldonado M., Spindler K.D., Eckert C., Hanke T., Born R., Goebel C., Simon P., Heinemann S., Worch H. (2007). First evidence of chitin as a component of the skeletal fibers of marine sponges. Part, I. Verongidae (demospongia: Porifera). J. Exp. Zool. B Mol. Dev. Evol..

[69] Ehrlich H., Krautter M., Hanke T., Simon P., Knieb C., Heinemann S., Worch H. (2007). First evidence of the presence of chitin in skeletons of marine sponges. Part II. Glass sponges (Hexactinellida: Porifera). J. Exp. Zool..

[70] Steck E., Burkhardt M., Ehrlich H., Richter W. (2010). Discrimination between cells of murine and human origin in xenotransplants by species specific genomic in situ hybridization. Xenotransplantation.

[71] Ehrlich H., Bates S.S., La Barre S. (2019). Chapter 26: Chitin of Poriferan Origin as a Unique Biological Material. Blue Biotechnol. Prod. Use Mar. Mol..

[72] Taylor D.A., Sampaio L.C., Ferdous Z., Gobin A.S., Taite L.J. (2018). Decellularized matrices in regenerative medicine. Acta Biomater..

[73] Rogulska O.Y., Mutsenko V.V., Revenko E.B., Petrenko Y.A., Ehrlich H., Petrenko A.Y. (2013). Culture and differentiation of human adipose tissue mesenchymal stromal cells within carriers based on sea sponge chitin skeletons. Probl. Cryobiol. Cryomedicine.

[74] Rogulska O.Y., Revenko O.B., Petrenko Y.O., Ehrlich H., Petrenko O.Y. (2013). Prospects for the application of Aplysinidae family marine sponge skeletons and mesenchymal stromal cells in tissue engineering. Biotechnologia Acta.

[75] Mutsenko V.V., Bazhenov V.V., Rogulska O., Tarusin D.N., Schütz K., Brüggemeier S., Gossla E., Akkineni A.R., Meissner H., Lode A. (2017). 3D chitinous scaffolds derived from cultivated marine demosponge *Aplysina aerophoba* for tissue engineering approaches based on human mesenchymal stromal cells. Int. J. Biol Macromol..

[76] Mutsenko V.V., Gryshkov O., Lauterboeck L., Rogulska O., Tarusin D.N., Bazhenov V.V., Schütz K., Brüggemeier S., Gossla E., Akkineni A.R. (2017). Novel chitin scaffolds derived from marine sponge *Ianthella basta* for tissue engineering approaches based on human mesenchymal stromal cells: Biocompatibility and cryopreservation. Int. J. Biol. Macromol..

[77] Mutsenko V., Gryshkov O., Rogulska O., Lode A., Petrenko A.Y., Gelinsky M., Glasmacher B., Ehrlich H., Choi A., Ben-Nissan B. (2019). Chitinous scaffolds from marine sponges for tissue engineering. Marine-Derived Biomaterials for Tissue Engineering Applications.

[78] Wagge L.E. (1951). The activity of amoebocytes and of alkaline phosphatases during the regeneration of the shell in the snail, *Helix aspersa*. Q. J. Microsc. Sci..

[79] Abolins-Krogis A. (1976). Ultrastructural study of the shell-repair membrane in the snail *Helix pomatia* L.. Cell Tissue Res..

[80] Mount A.S., Wheeler A.P., Paradkar R.P., Snider D. (2004). Hemocyte-mediated shell mineralization in the eastern oyster. Science.

[81] Chan V.B.S., Johnstone M.B., Wheeler A.P., Mount A.S. (2018). Chitin facilitated mineralization in the Eastern oyster. Front. Mar. Sci..

[82] Li S., Liu Y., Liu C., Huang J., Zheng G., Xie L., Zhang R. (2016). Hemocytes participate in calcium carbonate crystal formation, transportation *Pinctada fucata*. Fish. Shellfish Immunol..

[83] Mount A.S., Gohad N.V., Hansen D.C., Muller K., Johnstone M.B. (2013). Deposition of Nanocrystalline Calcite on Surfaces by A Tissue and Cellular Biomineralization. U.S. Patent.

[84] Mount A.S., Gohad N.V., Hansen D.C., Hansen K.M., Johnstone M.B. (2016). Deposition of nanocrystalline calcite on surfaces by a tissue and cellular biomineralization. U.S. Patent.

[85] Johnstone M.B., Gohad N.V., Falwell E.P., Hansen D.C., Hansen K.M., Mount A.S. (2015). Cellular orchestrated biomineralization of crystalline composites on implant surfaces by the eastern oyster, *Crassostrea virginica* (Gmelin, 1791). J. Exp. Mar. Bio. Ecol..

[86] Szkucik K., Ziomek M., Paszkiewicz W., Drozd Ł., Gondek M., Knysz P. (2018). Fatty acid profile in fat obtained from edible part of land snails harvested in Poland. J. Vet. Res..

[87] Biradar S., Ravichandran P., Gopikrishnan R., Goornavar V., Hall J.C., Ramesh V., Baluchamy S., Jeffers R.B., Ramesh G.T. (2011). Calcium carbonate nanoparticles: Synthesis, characterization and biocompatibility. J. Nanosci. Nanotechnol..

[88] Poologasundarampillai G., Boix-Alberich M., Clarke D., Smith A., Martin R., Lee P.D., Jones J.R. (2016). Hydroxyapatite or calcite: How does physiological proteins influence the type, mechanism and kinetics of their formation on bioactive glasses?. Front. Bioeng. Biotechnol..

[89] Bahrom H., Goncharenko A.A., Fatkhutdinova L.I., Peltek O.O., Muslimov A.R., Koval O.Y., Eliseev I.E., Manchev A., Gorin D., Shishkin I.I. (2019). Controllable Synthesis of Calcium Carbonate with Different Geometry: Comprehensive Analysis of Particle Formation, Cellular Uptake, and Biocompatibility. ACS Sustain. Chem. Eng..

[90] Fujita Y., Yamamuro T., Nakamura T., Kotani S., Ohtsuki C., Kokubo T. (1991). The bonding behavior of calcite to bone. J. Biomed. Mater. Res..

[91] Geblinger D., Geiger B., Addadi L. (2009). Surface-induced regulation of podosome organization and dynamics in cultured osteoclasts. ChemBioChem.

[92] Geblinger D., Addadi L., Geiger B. (2010). Nano-topography sensing by osteoclasts. J. Cell Sci..

[93] Lopez E., Vidal B., Berland S., Camprasse S., Camprasse G., Silve C. (1992). Demonstration of the capacity of nacre to induce bone formation by human osteoblasts maintained in vitro. Tissue Cell.

[94] Fricain J.C., Bareille R., Ulysse F., Dupuy B., Amedee J. (1998). Evaluation of proliferation and protein expression of human bone marrow cells cultured on coral crystallized in the aragonite of calcite form. J. Biomed. Mater. Res..

[95] Kreklau B., Sittinger M., Mensing M.B., Voigt C., Berger G., Burmester G.R., Rahmanzadeh R., Gross U. (1999). Tissue engineering of biphasic joint cartilage transplants. Biomaterials.

[96] Demers C., Hamdy C.R., Corsi K., Chellat F., Tabrizian M., Yahia L. (2002). Natural coral exoskeleton as a bone graft substitute: a review. Biomed. Mater. Eng..

[97] Michalowski S., Jaegermann Z., Karas J. (2004). Properties of calcite materials for cell culture scaffolds. EngBiomater..

[98] Rocha J.H., Lemos A.F., Agathopoulos S., Valerio P., Kannan S., Oktar F.N., Ferreira J.M. (2005). Scaffolds for bone restoration from cuttlefish. Bone.

[99] Liu Y., Jiang T., Zhou Y., Zhang Z., Wang Z.J., Tong H., Shen X.Y., Wang Y.N. (2011). Evaluation of the attachment, proliferation, and differentiation of osteoblast on a calcium carbonate coating on titanium surface. Mater. Sci. Eng. C..

[100] Monchau F., Hivart P., Genestie B., Chai F., Descamps M., Hildebrand H.F. (2013). Calcite as a bone substitute. Comparison with hydroxyapatite and tricalcium phosphate with regard to the osteoblastic activity. Mater. Sci. Eng. C.

[101] Kamba A.S., Zakaria Z.A.B. (2014). Osteoblasts growth behaviour on bio-based calcium carbonate aragonite nanocrystal. BioMed Res. Int..

[102] Tolba E., Müller W.E.G., Abd El-Hady B.M., Neufurth M., Wurm F., Wang S., Schröder H.C., Wang X. (2016). High biocompatibility and improved osteogenic potential of amorphous calcium carbonate/vaterite. J. Mater. Chem. B.

[103] Chróścicka A., Jaegermann Z., Wychowański P., Ratajska A., Sadło J., Hoser G., Michałowski S., Lewandowska-Szumiel M. (2016). Synthetic Calcite as a Scaffold for Osteoinductive Bone Substitutes. Ann. Biomed. Eng..

[104] Woldetsadik A.D., Sharma S.K., Khapli S., Jagannathan R., Magzoub M. (2017). Hierarchically Porous Calcium Carbonate Scaffolds for Bone Tissue Engineering. ACS Biomater. Sci. Eng..

[105] Douglas T.E.L., Łapa A., Samal S.K., Declercq H.A., Schaubroeck D., Mendes A.C.L., Van der Voort P., Dokupil A., Plis A., De Schamphelaere K. (2017). Enzymatic, urease-mediated mineralization of gellan gum hydrogel with calcium carbonate, magnesium-enriched calcium carbonate and magnesium carbonate for bone regeneration applications. J. Tiss. Eng. Regen. Med..

[106] Higgin T. (1875). On a new sponge of the genus *Luffaria*, from Yucatan, in the Liverpool Free Museum. Ann. Mag. Nat. Hist..

[107] Villamizar E., Laughlin R.A. (1991). Fauna Associated with the Sponges *Aplysina archeri* and *Aplysina lacunosa* in a Coral Reef of the Archipiélago de Los Roques, National Park, Venezuela. Fossil and Recent Sponges.

[108] Humann P., DeLoach N. (2002). Reef Creature Identification: Florida, Caribbean, Bahamas.

[109] Alcolado P.M., Busutil L. (2012). Inventaire des spongiaires néritiques du Parc National de La Guadeloupe. Serie Oceanológica.

[110] Yoshino T.P., Bickham U., Bayne C.J. (2013). Molluscan cells in culture: Primary cell cultures and cell lines. Can. J. Zool..

[111] Grandiosa R., Mérien F., Pillay K., Alfaro A. (2016). Innovative application of classic and newer techniques for the characterization of haemocytes in the New Zealand black-footed abalone (*Haliotis iris*). Fish. Shellfish Immunol..

[112] Fernández M.S., Valenzuela F., Arias J.I., Neira-Carrillo A., Arias J.L. (2016). Is the snail shell repair process really influenced by eggshell membrane as a template of foreign scaffold?. J. Struct. Biol..

[113] Huang J., Li S., Liu Y., Liu C., Xie L., Zhang R. (2018). Hemocytes in the extrapallial space of *Pinctada fucata* are involved in immunity and biomineralization. Sci. Rep..

[114] Modreski P.J., Aumente-Modreski R. (1996). Fluorescent minerals, a review. Rocks Miner. Mag..

[115] Kumirska J., Czerwicka M., Kaczyński Z., Bychowska A., Brzozowski K., Thöming J., Stepnowski P. (2010). Application of spectroscopic methods for structural analysis of chitin and chitosan. Mar. Drugs.

[116] Dement J. (2013). Handbook of Fluorescent Gems and Minerals—An Exposition and Catalog of the Fluorescent and Phosphorescent Gems and Minerals, Including the Use of Ultraviolet Light in the Earth Sciences.

[117] Reig F.B., Adelantado J.V.G., Moya Moreno M.C.M. (2002). FTIR quantitative analysis of calcium carbonate (calcite) and silica (quartz) mixtures using the constant ratio method. Application to geological samples. Talanta.

[118] Coleyshaw E.E., Crump G., Griffith W.P. (2003). Vibrational spectra of the hydrated carbonate minerals ikaite, monohydrocalcite, lansfordite and nesquehonite. Spectrochim. Acta. A. Mol. Biomol. Spectrosc..

[119] Prinsloo L.C. (2007). Rock hyraces: A cause of San rock art deterioration?. J. Raman Spectrosc..

[120] Señorale-Pose M., Chalar C., Dauphin Y., Massard P., Pradel P., Marín M. (2008). Monohydrocalcite in calcareous corpuscles of *Mesocestoides corti*. Exp. Parasitol..

[121] Iordanidis A., Garcia-Guinea J., Strati A., Gkimourtzina A., Papoulidou A. (2011). Thermal, mineralogical and spectroscopic study of plasters from three post-Byzantine churches from Kastoria (northern Greece). J. Therm. Anal. Calorim..

[122] Singh N., Koziol K.K.K., Chen J., Patil A.J., Gilman J.W., Trulove P.C., Kafienah W., Rahatekar S.S. (2013). Ionic liquids-based processing of electrically conducting chitin nanocomposite scaffolds for stem cell growth. Green Chem..

[123] Wehrmeister U., Jacob D.E., Soldati A.L., Häger T., Hofmeister W. (2007). Vaterite in freshwater cultured pearls from China and Japan. J. Gemmol..

[124] Bischoff W.D., Sharma S.K., MacKenzie F.T. (1985). Carbonate ion disorder in synthetic and biogenic magnesian calcites: a Raman spectral study. Am. Mineral..

[125] Kadokawa J.I. (2015). Fabrication of nanostructured and microstructured chitin materials through gelation with suitable dispersion media. RSC Adv..

[126] Silva S.S., Mano J.F., Reis R.L. (2017). Ionic liquids in the processing and chemical modification of chitin and chitosan for biomedical applications. Green Chem..

[127] Muzzarelli R.A.A. (2011). Biomedical exploitation of chitin and chitosan via mechano-chemical disassembly, electrospinning, dissolution in imidazolium ionic liquids, and supercritical drying. Mar. Drugs.

[128] Muzzarelli R.A.A., El Mehtedi M., Mattioli-Belmonte M. (2014). Emerging biomedical applications of nano-chitins and nano-chitosans obtained via advanced eco-friendly technologies from marine resources. Mar. Drugs.

[129] Coltelli M.B., Gigante V., Panariello L., Morganti P., Cinelli P., Danti S., Lazzeri A. (2018). Chitin nanofibrils in renewable materials for packaging and personal care applications. Adv. Mater. Lett..

[130] Coltelli M.-B., Cinelli P., Gigante V., Aliotta L., Morganti P., Panariello L., Lazzeri A. (2019). Chitin Nanofibrils in Poly (Lactic Acid) (PLA) Nanocomposites: Dispersion and Thermo-Mechanical Properties. Int. J. Mol. Sci..

[131] Pina S., Ribeiro V.P., Marques C.F., Maia R., Silva T.H., Reis R.L., Oliveira J.M. (2019). Scaffolding Strategies for Tissue Engineering and Regenerative Medicine Applications. Materials.

[132] Chocholata P., Kulda V., Babuska V. (2019). Fabrication of scaffolds for bone-tissue regeneration. Materials.

[133] Cheng T.C. (1984). A Classification of Molluscan Hemocytes Based on Functional Evidences. Invertebrate Blood.

[134] Cheng T.C. (1975). Functional morphology and biochemistry of molluscan phagocytes. Ann. N. Y. Acad. Sci..

[135] Cheng T.C., Guida V.G. (1980). Hemocytes of *Bulinus truncatus rohlfsi* (Mollusca: Gastropoda). J. Invertebr. Pathol..

[136] Adema C.M., Harris R.A., van Deutekom-Mulder E.C. (1992). A comparative study of hemocytes from six different snails. J. Invertabrates Pathol..

[137] Adamowicz A., Bolaczek M. (2003). Blood cells morphology of the snail *Helix aspersa maxima* (Helicidae). Zool. Pol..

[138] Bayne C.J., Moore M.N., Carefoot T.H., Thompson R.J. (1979). Hemolymph functions in *Mytilus californianus*: The cytochemistry of hemocytes and their responses to foreign implants and hemolymph factors in phagocytosis. J. Invertebr. Pathol..

[139] Pila E.A., Sullivan J.T., Wu X.Z., Fang J., Rudko S.P., Gordy M.A., Hanington P.C. (2016). Haematopoiesis in molluscs: a review of haemocyte development and function in gastropods, cephalopods and bivalves. Dev. Comp. Immunol..

[140] Sminia T., Van der Knaap W.P.W., Van Asselt L.A. (1983). Blood cell types and blood cell formation in gastropod molluscs. Dev. Comp. Immunol..

[141] Sminia T., Barendsen L. (1980). A comparative morphological and enzyme histochemical study on blood cells of the freshwater snails *Lymnae astagnalis*, *Biomphalaria glabrata*, and *Bulinus truncatus*. J. Morphol..

[142] Addadi L., Joester D., Nudelman F., Weiner S. (2006). Mollusk shell formation: a source of new concepts for understanding biomineralization processes. Chem. - A Eur. J..

[143] Du X., Fan G., Jiao Y., Zhang H., Guo X., Huang R., Zheng Z., Bian C., Deng Y., Wang Q. (2017). The pearl oyster *Pinctada fucata martensii* genome and multi-omic analyses provide insights into biomineralization. Gigascience.

[144] Abolins-Krogis A. (1973). Fluorescence and histochemical studies of the calcification-initiating lipofuscin type pigment granules in the shell-repair membrane of the snail, *Helix pomatia* L.. Zeitschrift fur Zellforsch. und mikroskopische Anat..

[145] Xiang L., Kong W., Su J., Liang J., Zhang G., Xie L., Zhang R. (2014). Amorphous calcium carbonate precipitation by cellular biomineralization in mantle cell cultures of *Pinctada fucata*. PLoS One.

[146] Kapur S.P., Sen Gupta A. (1970). The role of amoebocytes in the regeneration of shell in the land pulmonate, *Euplecta indica* (Pfieffer). Biol. Bull..

[147] Abolins-Krogis A. (1968). Shell regeneration in *Helix pomatia* with special reference to the elementary calcifying particles. Symp. Zool. Soc. London.

[148] Abolins-Krogis A. (1963). The histochemistry of the mantle of *Helix pomatia* (L.) in relation to the repair of the damaged shell. Ark. för Zool..

[149] Munro N.H., McGrath K.M. (2012). Biomimetic approach to forming chitin/aragonite composites. Chem. Commun..

[150] Falini G., Fermani S., Ripamonti A. (2002). Crystallization of calcium carbonate salts into beta-chitin scaffold. J. Inorg. Biochem..

[151] Sommerdijk N.A.J.M., With G. (2008). de Biomimetic CaCO_3_ Mineralization using Designer Molecules and Interfaces. Chem. Rev..

[152] Xu Y., Tijssen K.C.H., Bomans P.H.H., Akiva A., Friedrich H., Kentgens A.P.M., Sommerdijk N.A.J.M. (2018). Microscopic structure of the polymer-induced liquid precursor for calcium carbonate. Nat. Commun..

[153] Kayano K., Saruwatari K., Kogure T., Shiraiwa Y. (2011). Effect of Coccolith Polysaccharides Isolated from the Coccolithophorid, *Emiliania huxleyi*, on Calcite Crystal Formation in in vitro CaCO_3_ Crystallization. Mar. Biotechnol..

[154] Matsumura S., Kajiyama S., Nishimura T., Kato T. (2015). Formation of Helically Structured Chitin/CaCO_3_ Hybrids through an Approach Inspired by the Biomineralization Processes of Crustacean Cuticles. Small.

[155] Gal A., Wirth R., Barkay Z., Eliaz N., Scheffel A., Faivre D. (2017). Templated and self-limiting calcite formation directed by coccolith organic macromolecules. Chem. Commun..

[156] Liu M.F., Lu Z., Zhang Z., Xiao C., Li M., Huang Y.X., Liu X.Y., Jiang Y. (2018). Correlations of crystal shape and lateral orientation in bioinspired CaCO_3_ mineralization. CrystEngComm.

[157] Kruppke B., Farack J., Weil S., Aflalo E.D., Poláková D., Sagi A., Hanke T. (2019). Crayfish hemocyanin on chitin bone substitute scaffolds promotes the proliferation and osteogenic differentiation of human mesenchymal stem cells. J. Biomed. Mater. Res..

[158] Astachov L., Nevo Z., Vago R. (2012). Calcite Biohybrids as Microenvironment for Stem Cells. Polymers.

[159] Ziętek J., Guz L., Panasiuk K., Winiarczyk S., Adaszek Ł. (2017). Nowa metoda przyżyciowego pobierania hemolimfy wraz z ustaleniem norm fizjologicznych wybranych parametrów biochemicznych hemolimfy ślimaków *Cornu aspersum*. Med. Weter..

[160] Saleuddin A.S.M., Chan W. (1969). Shell regeneration in *Helix*: shell matrix composition and crystal formation. Can. J. Zool..

[161] Abolins-Krogis A. (1958). The morphological and chemical characteristics of organic crystals in the regenerating shell of *Helix pomatia* L.. Acta Zool..

[162] De Waele A. (1930). Le sang d’Anodonta cygnea et la formation de la coquille. Bull. l’Académie R. des Sci. Belgique Cl. des Sci..

[163] Lebel J.-M., Giard W., Favrel P., Boucaud-Camou E. (1996). Effects of different vertebrate growth factors on primary cultures of hemocytes from the gastropod mollusc, *Haliotis tuberculata*. Biol Cell.

[164] Donaghy L., Hong H.K., Lambert C., Park H.S., Shim W.J., Choi K.S. (2010). First characterisation of the populations and immune-related activities of hemocytes from two edible gastropod species, the disk abalone, *Haliotis discus discus* and the spiny top shell, *Turbo. cornutus*. Fish. Shellfish Immunol..

[165] Li M., He P., Wu Y., Zhang Y., Xia H., Zheng Y., Han Y. (2016). Stimulatory effects of the degradation products from Mg-Ca-Sr alloy on the osteogenesis through regulating ERK signaling pathway. Sci. Rep..

